# Pleiotropy and synergistic cooperation

**DOI:** 10.1371/journal.pbio.3000320

**Published:** 2019-06-21

**Authors:** David C. Queller

**Affiliations:** 1 Department of Biology, Washington University in St. Louis, St. Louis, Missouri, United States of America; 2 Wissenschaftskolleg zu Berlin, Berlin, Germany

## Abstract

Some forms of stable cooperation can evolve though pleiotropy with a beneficial private trait. This Formal Comment addresses a recent challenge to this idea, arguing that for synergistic, frequency-dependent cooperation, pleiotropy can raise the frequency up to a point where cooperation is favoured on its own.

Costly cooperation can be difficult to evolve because noncooperators or cheaters do not pay the cost but reap the rewards of their neighbors’ cooperation. One suggested mechanism is for cooperation to be associated, by pleiotropy or linkage, with some private beneficial trait [1] that noncooperators lack. Despite a number of putative examples, dos Santos and colleagues [2] argue that such cooperation is not stable, because the traits can eventually be dissociated by changes in regulation or by recombination.

The argument is an example of Hammerstein’s [3] "streetcar" theory. He argued that models with genetic details—in this case pleiotropy or linkage—can sometimes mislead, given that these details can change. Though one could argue that some constraints may be permanent, this is a reasonable argument against pleiotropy permanently stabilizing cooperation.

But pleiotropy or linkage can be crucial in a different and unappreciated way for a large class of cooperative behaviors—those with positive frequency-dependent selection owing to positive synergistic selection. Fig 1a shows a simple example, in which an individual’s (inclusive) fitness depends of the frequency of the cooperative trait in the population. In positive frequency-dependent selection, fitness increases with frequency, so in some cases, an allele is favored only if it can become sufficiently common, crossing the frequency threshold beyond which fitness is positive.

**Fig 1:**
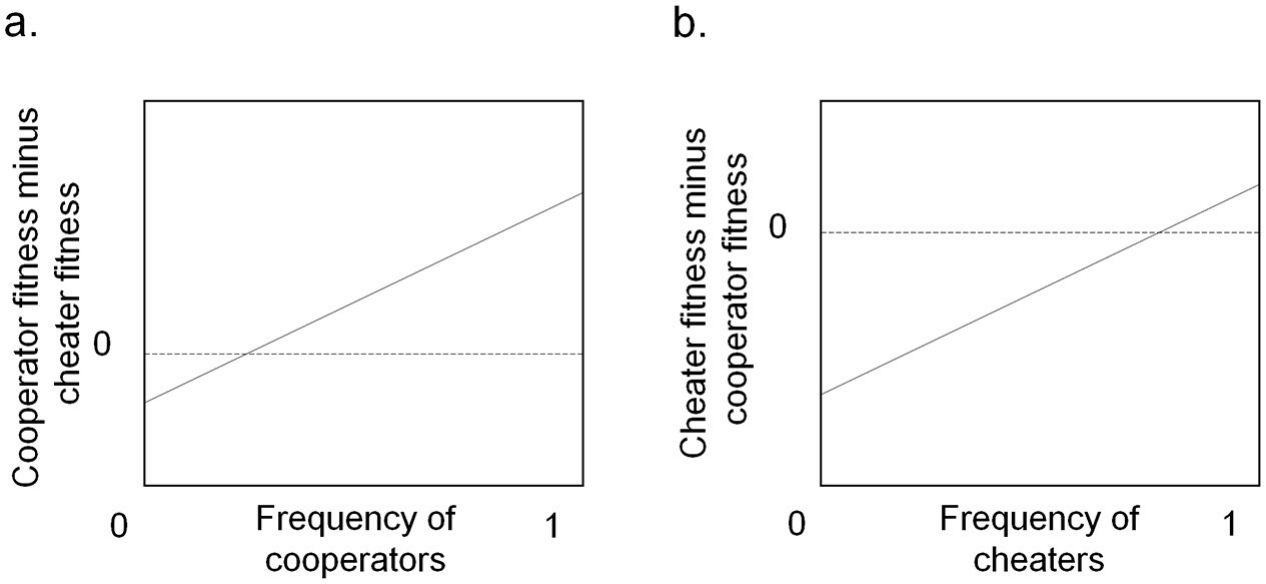
Positive frequency dependence. (a) Cooperator point of view. Cooperators become more fit than cheaters only above a threshold frequency of cooperators. (b) Cheater point of view (the same graph rotated 180° to reverse the axes). Cheaters become more fit than cooperators only above a threshold frequency of cheaters.

Here, the possible role for pleiotropy or linkage is simple. A mutant allele that is both cooperative and associated by linkage or pleiotropy with a sufficiently beneficial private trait will increase in frequency. The association may ultimately be temporary, but if selection has pushed the cooperation gene across the fitness threshold, the association is no longer needed; the cooperation allele is then favored on its own merits. And because both traits are favorable at this point, there will be no selection for breaking the association.

Frequency-dependent selection occurs because of nonadditive fitness interactions [4]. I have called this "kind selection" because the effect of one’s own behavior depends on the kind of partner one has [5]. In the simplest case, suppose a cooperator loses *c* additive fitness units from its own behavior, gains an additive *b* units if its partner cooperates, and if both cooperate, gets a further *d* units, a nonadditive or synergistic term. This results in a version of Hamilton’s rule that says cooperation is favored when −*c* + *rb* + *sd*, where *r* is relatedness and *s* is a synergism coefficient that is an increasing function of both relatedness and population frequency [4, 5]. For the case in which the behavior is caused by a single gene with complete penetrance, $s\;=\;r\;+(1-r)\;\bar{p}$, where $\bar{p}$ is the population frequency of the cooperation allele and trait [4]. Thus, even at a low frequency, a positive *r* can make the synergistic effect positive and select for cooperation. (For a low-penetrance allele whose partners will not express cooperation, $s\;=\;\bar{p}$, so such invasion will not work, but once a high-penetrance allele makes the frequency of cooperators $\bar{p}$ high, then a low-penetrance allele also favors cooperation, so cooperation will be stable).

Some versions of Hamilton’s rule, such as those using partial regression coefficients for *b* and *c*, assimilate the synergism term into the cost and benefit terms [6]. This is legitimate and useful for some purposes, but folding the frequency dependence into the partial regressions does tend to obscure it. Breaking out the synergistic frequency dependence reminds us to think about its special effect.

Such synergism must be quite common. The entire application of game theory to biology rests on either positive or negative synergistic effects (or similar nonadditivities in more complex models, such as with multiple players [7]). For example, in the classic hawk–dove game (or hawk–mouse [8]), doves cooperatively share a resource instead of fighting over it, but being a dove doesn’t pay unless they are at sufficiently high frequency. In real biology, positive synergism emerges from common phenomena, including division of labor and economies of scale [9].

Dos Santos and colleagues [2] noted that cheaters could also benefit from an association with a beneficial private trait. This remains a good point under positive frequency dependence. Fig 1b illustrates this by rotating Fig 1a 180°, reversing the axes to give the cheater’s point of view for the same fitness parameters. Just like cooperators, cheaters win only if they are sufficiently common, and they might reach that threshold via association with a private advantage. For the particular line in Fig 1, it is easier for a cooperator to reach its threshold frequency than it is for a cheater, though the reverse can be true for other lines. But noncooperation is generally the default state and is therefore not viewed as needing any special explanation. Cooperation does need special explanations, and the pleiotropy-linkage argument, coupled with synergism, gives one such explanation.

In summary, although pleiotropy and linkage may not usually stabilize cooperation in the long term [2], they can be important in a different way. When they can pull synergistic cooperation to the point at which it is stable on its own, then it doesn’t matter if the pleiotropy and linkage break down, because the cooperation is stabilized by there being many cooperators. Moreover, there would be no selection for such a breakdown, so we might expect to observe a continuing association in nature between cooperation and beneficial nonsocial traits. However, the model makes the testable prediction that any such association in nature will primarily involve positively synergistic cooperation.

## References

[1] Foster KR, Shaulsky G, Strassmann JE, Queller DC, Thompson CRL. Pleiotropy as a mechanism to stabilize cooperation. Nature. 2004;431:693–6. 10.1038/nature02894 15470429

[2] Dos Santos M, Ghoul M, West SA. Pleiotropy, cooperation, and the social evolution of genetic architecture. PLoS Biol. 2018;16:e2006671 10.1371/journal.pbio.2006671 30359363 PMC6219813

[3] Hammerstein P. Darwinian adaptation, population genetics and the streetcar theory of evolution. J Math. Biol. 1996;34:511–32. 8691083 10.1007/BF02409748

[4] Queller DC. Kin selection and frequency dependence: a game theoretic approach. Biol. J. Linnean Soc. 1984;23:133–43.

[5] Queller DC. Expanded social fitness and Hamilton’s rule for kin, kith, and kind. Proc. Nat. Acad. Sci. USA. 2011;108:10792–9. 10.1073/pnas.1100298108 21690389 PMC3131824

[6] Queller DC. A general model for kin selection. Evolution. 1992;46:376–80. 10.1111/j.1558-5646.1992.tb02045.x 28564031

[7] Hauert C, Michor F, Nowak MA, Doebeli M. Synergy and discounting of cooperation in social dilemmas. J. Theor. Biol. 2006;239:195–202. 10.1016/j.jtbi.2005.08.040 16242728 PMC2891160

[8] Maynard Smith J, Price GR. The logic of animal conflict. Nature. 1973;246:15–8.

[9] Corning PA, Szathmáry E. “Synergistic selection”: A Darwinian frame for the evolution of complexity. J. Theor. Biol. 2015;371:45–58. 10.1016/j.jtbi.2015.02.002 25681798

